# Reproduction of *Meloidogyne arenaria* race 2 on flue-cured tobacco with putative resistance derived from *Nicotiana repanda*

**DOI:** 10.21307/jofnem-2021-064

**Published:** 2021-07-19

**Authors:** Noah Adamo, Charles S. Johnson, T. David Reed, Jonathan D. Eisenback

**Affiliations:** 1 Virginia Tech, Southern Piedmont Agricultural Research and Extension Center, 2375 Darvills Rd, Blackstone, VA, 23824; 2 Virginia Tech, School of Plant and Environmental Sciences, 170 Drillfield Dr, Blacksburg, VA, 24060

**Keywords:** *Meloidogyne arenaria*, *Nicotiana tabacum*, *Nicotiana repanda*, Flue-cured tobacco, Resistance, Management

## Abstract

Chemical controls for root-knot nematodes are increasingly restricted due to environmental and human health concerns. Host resistance to these nematodes is key to flue-cured tobacco production in Virginia. Resistance to *Meloidogyne incognita* races 1 and 3, and race 1 of *M. arenaria* is imparted by the gene *Rk1,* which is widely available in commercial flue-cured tobacco. *Rk2* imparts increased resistance to *M. javanica* when stacked with *Rk1* and is becoming commercially available. The efficacy of *Rk2* against *M. arenaria* race 2, which is increasingly prevalent in Virginia, is unclear. Greenhouse trials were conducted in 2017 to determine how potential resistance derived from *N. repanda* compares to the root-knot nematode resistance afforded by *Rk1* and *Rk2*. Trials were arranged in a completely randomized block design and included an entry with traits derived from *N. repanda*, a susceptible entry and entries possessing *Rk1* and/or *Rk2*. Data collected after 60 days included percent root galling, egg mass counts, and egg counts. Root galling and reproduction were significantly lower on the entry possessing traits derived from *N. repanda* relative to other entries, suggesting that the *N. repanda* species may hold a novel source of root-knot nematode resistance for commercial flue-cured tobacco cultivars.

Tobacco (*Nicotiana tabacum* L.) is a valuable agricultural commodity cultivated around the world, with the value of unprocessed tobacco globally estimated at over 17 billion dollars in 2018 (FAO, 2018). Tobacco production has declined considerably in the United States over the past two decades, but unprocessed tobacco sales still generated nearly 1.1 billion dollars nationally as of 2018 (FAO, 2018). In the United States, flue-cured tobacco accounts for the majority of the crop, with over 5,660 hectares planted in Virginia alone in 2020 (USDA, 2020). Root-knot nematodes (*Meloidogyne* spp.) can seriously complicate production for tobacco growers in the southeastern United States, causing crop losses of 1 to 5% in Virginia in affected fields (Fortnum et al., 2001; Koenning et al., 1999). The use of resistant cultivars for root-knot nematode management is a fundamental tool for flue-cured tobacco growers (Johnson et al., 2005), particularly in light of the increasing restrictions on some of the most historically effective chemical management options, including soil fumigants (USEPA, 2008) and non-fumigant nematicides in the organophosphate and carbamate classes (USEPA, 2020).

Resistance is defined in plant nematology as the near or complete inhibition of reproduction by the nematode, whereas tolerance is defined as a response in which nematode reproduction is not necessarily inhibited, but in which the host does not suffer serious deleterious consequences as a result of nematode parasitism and reproduction (Roberts, 2002). In flue-cured tobacco, resistance to root-knot nematodes is mediated by one or both of two genes. Root-knot nematode resistance first became commercially available in Zimbabwe in 1961 with the introduction of a cultivar possessing a gene, *Rk*, originally discovered in *N. tomentosa* Ruis and Pav (Yi et al., 1998). This gene, now known as *Rk1*, is widely available in commercial flue-cured tobacco cultivars and imparts what has been described as some level of resistance to *M. incognita* (Kofoid and White) Chitwood (1949) host races 1 and 3 and *M. arenaria* (Neal) Chitwood (1949) host race 1 (Ng’ambi et al., 1999b; Schneider, 1991). *Rk1* may have some inhibitory effect on the reproduction of *M. javanica*on flue-cured tobacco (Ternouth et al., 1986) but contradicting research suggests that *Rk1* imparts minimal or no resistance to *M. javanica*, *M. incognita* host races 2 and 4, *M. arenaria* race 2, and *M. hapla* Chitwood (1949) (Ng’ambi et al., 1999b).

In 1993, another root-knot nematode resistance gene, now known as *Rk2*, was introduced into a cultivar commercially available in Zimbabwe, Kutsaga RK26, stacked with *Rk1* (Jack, 2001; Jack and Lyle, 1999; Way, 1994a). This gene, originally known as ‘*T*,’ was discovered in 1950 in *N. tabacum* plants in subsistence gardens along the Zambezi river in Zimbabwe. These gardens had been maintained for over 250 years in soils heavily infested with *M. javanica* (Mackenzie et al., 1986; Schweppenhauser, 1975; Ternouth et al., 1986). This gene imparts resistance to *M. javanica* and imparts far greater resistance to this nematode than *Rk1,* although combining both genes resulted in an even higher level of resistance (Schweppenhauser, 1975; Ternouth et al., 1986). Tobacco cultivars possessing both *Rk1* and *Rk2* became commercially available in the United States from Cross Creek Seed Company and ProfiGen do Brasil in 2007 (Reed, 2007).

Recent research has confirmed the high level of resistance conferred by the combination of both *Rk1* and *Rk2* to populations of *M. javanica* (Johnson, unpublished data) and *M. incognita* race 3 (Pollok et al., 2016) from Virginia flue-cured tobacco fields. *Meloidogyne incognita* had historically been regarded as the greatest nematode threat to tobacco in Virginia (Johnson, 1989), but over the past several decades, *M. arenaria* has superseded *M. incognita* in abundance (Eisenback, 2012). A 2004 survey of 170 Virginia tobacco fields found 43.5% of surveyed fields were infested with root-knot nematodes, with *M. arenaria* present in 56.7% of infested fields and *M. incognita* present in 16.7% of infested fields, while *M. hapla, M. javanica*, and unidentified root-knot species were present in 25, 11.7, and 8.3% of infested fields, respectively (Eisenback, 2012). In a 2010 survey of 276 Virginia flue-cured tobacco fields, root-knot nematodes were present in 44.9% of surveyed fields, with *M. arenaria* present in 58.8% of those infested fields, while *M. incognita* was present in only 11.1% of infested fields, with other species present at similar levels to 2004 (Eisenback, 2012).

*Nicotiana repanda* has long been seen as a potential source of disease resistance for cultivated tobacco, including root-knot nematode resistance (Burk and Heggestad, 1966; Stavely et al., 1973). Schweppenhauser (1975) observed that *N. repanda* was the only species evaluated out of 64 *Nicotiana* species that was completely resistant to *M. javanica*. However, due to chromosomal incompatibilities between *N. repanda* and *N. tabacum*, the manifestation of lethal genes, and other factors, attempts to hybridize or incorporate genes from *N. repanda* with commercially viable *N. tabacum* selections were not successful for many years (Bui et al., 1992). In the late 1960s, and again in the mid 1980s, attempts were made to integrate resistance to tobacco mosaic virus (TMV), tobacco cyst nematode (*Globodera tabacum solanacearum,* Miller and Gray) and *M. javanica* and *M. arenaria* from *N. repanda* into two commercial tobacco cultivars using an interspecific bridge hybridization procedure (Burk, 1967; Gwynn et al., 1986). These crosses were ultimately successful in transferring TMV and cyst nematode resistance to some progeny lines, but root-knot nematode resistance was lost after necessary backcrosses (Davis et al., 1988a, 1988b). Cell culture techniques were also employed with little success (Bui et al., 1992). However, resistance to *M. arenaria* and TMV was successfully transferred from *N. repanda* to *N. tabacum* in 1992 using *N. sylvestris* as a bridge species, along with a form of protoplast fusion (Bui et al., 1992). The objective of this work was to evaluate resistance to a population of *M. arenaria* race 2 in a flue-cured tobacco entry possessing traits from *N. repanda,* and to compare the resistance of this entry to that of flue-cured tobacco entries possessing the root-knot nematode resistance genes *Rk1* and/or *Rk2* alone or in combination.

## Materials and methods

### Greenhouse trials

A nematode population originally obtained from a flue-cured tobacco field in Halifax County, Virginia provided inoculum in these trials. The population was maintained on susceptible tomato (*Solanum lycopersicum* L.) variety ‘Rutgers’ in greenhouse facilities on the Virginia Tech campus in Blacksburg, Virginia, and at the Virginia Tech Southern Piedmont Agricultural Research and Extension Center (SPAREC) in Blackstone, Virginia. The species and race identity had been previously confirmed as *M. arenaria* race 2 by morphological examination and differential host testing (Taylor and Sasser, 1978) and was verified by examination of more than 40 perineal patterns from the population (Eisenback, 1985). Egg inoculum for greenhouse trials was extracted from infested roots following the method of Hussey and Barker (1973). Four greenhouse experiments were conducted in 2017 to evaluate the impact of a putative source of resistance to root-knot nematodes derived from *N. repanda* on the reproductive capacity of a population of *M. arenaria* race 2 on a panel of seven flue-cured tobacco entries: Hicks (susceptible to four widely distributed root-knot nematode species, *M. incognita, M. javanica, M. arenaria,* and *M. hapla*); K326 (homozygous for *Rk1*); T-15-1-1 (homozygous for *Rk2*); CC 13 (homozygous for *Rk1* and heterozygous for *Rk2*); STNCB-2-28 (homozygous for both *Rk1* and *Rk2*); BAG 29-15-3-32-1 (also homozygous for both *Rk1* and *Rk2*); and 81-R-617A (contains traits derived from *N. repanda* which may influence resistance to root-knot nematodes). Seed of K326 and CC 13 were obtained from Cross Creek Seed (Raeford, NC). The seed of BAG 29-15-3-32-1 and 81-R-617A were provided by Dr. Ramsey Lewis at North Carolina State University (Raleigh, NC). Seed of the remaining four entries were produced in the flue-cured tobacco nursery at SPAREC. Trials were conducted concurrently in greenhouse facilities on the Virginia Tech campus in Blacksburg and at SPAREC in Blackstone from mid-June to mid-August and from early September to early November. Seed was germinated in organic vermiculite (The Epsoma Company, Millville, NJ). Four to five-week-old seedlings were transplanted to individual 7.6 cm diameter clay pots containing a 2:1 mixture of steam sterilized sandy loam field soil to Profile Greens Grade Porous Ceramic soil amendment (Profile Products, Buffalo Grove, IL). Seedlings with 4 to 6 true leaves were transplanted into 15 cm clay pots containing the same soil substrate. Plants were inoculated with a 40 ml aliquot containing 5,000 nematode eggs applied directly to the root mass during transplanting. Plants were maintained in greenhouses in Blacksburg at ambient temperature (27 ± 4°C) with natural lighting for the duration of the experiments. In trials conducted in Blackstone, plants were grown in open-top root zone growth chambers (Environmental Growth Chambers, Chagrin, OH) maintaining a soil temperature of ~27 ± 2°C (day/night cycle fluctuation) with ambient lighting inside of a larger greenhouse (air temperatures 27 ± 4°C).

Trials were terminated 60 days after inoculation and plants were evaluated for percent root galling, egg mass deposition, and egg production. The percent galled roots were estimated visually on the entire root mass of the plant. Root systems were subsequently cut into 4 to 6 cm sections and mixed thoroughly. Three 1 g subsamples were stained with 0.15 g/L Phloxine-B (Daykin and Hussey, 1985) for approximately 5 min to define egg masses and subsequently counted at ×10-×20 with a dissecting microscope. Eggs were extracted using the bleach agitation method of Hussey and Barker (1973) as described above. Eggs were suspended in 1 L of tap water and counted in two 10 ml aliquots counted at ×40 using an inverted compound microscope. Additionally, the reproductive index (*P*_*f*_/*P*_*i*_) was calculated for each plant by dividing the final egg count (*P*_*f*_) by the initial egg inoculum number (*P*_*i*_).

### Statistical analysis

Data from all trials were transformed (log_10_ (*x* + 1), or arcsine in the case of percent data) for analysis of variance (ANOVA) using PROC GLM in SAS (version 9.4; SAS Institute, Cary, NC). Differences among treatment means were identified using Fisher’s protected least significant difference test (*p* ≤ 0.05).

## Results

Both time and location of tests, and the interaction between test and genotype, had significant effects on results, so root-knot parasitism data from each trial were analyzed independently (*p* ≤ 0.0001 time and location; *p* ≤ 0.0181 interaction between test and genotype).

Significant differences in root galling were found among entries in all trials (Table 1). 81-R617A had significantly lower root galling than all other entries in the June to August trial conducted in Blackstone (Table 1). In the trial in Blacksburg from September to November, 81-R-617A exhibited significantly less root galling than all entries with the exception of STNCB 2-28 and BAG 29-15-3-32-1, both of which are homozygous for resistance genes *Rk1* and *Rk2* (Table 1). Similarly, in the trial in Blacksburg from June to August, galling was lowest on 81-R-617A and BAG 29-15-3-32-1 relative to T-15-1-1, which in this trial experienced the most root galling observed across all four trials (Table 1). Galling was significantly lower on 81-R-617A than on Hicks in three of the four trials. No entry consistently exhibited the highest levels of root galling in these trials (Table 1).

**Table 1.** Root galling of flue-cured tobacco entries by *Meloidogyne arenaria* race 2 in greenhouse pot tests in 2017^*^.

		Root % galling			
		June-August		September-November	
Genotype	Entry	Blackstone	Blacksburg	Blackstone	Blacksburg
*rk1rk2*	Hicks	42.5bc	42.5ab	45.0a	54.0a
*Rk1rk2*	K326	45.0bc	56.3ab	51.7a	21.0b
*rk1Rk2*	T-15-1-1	53.8abc	71.3a	36.7ab	21.0b
*Rk1Rk2*	CC 13	60.0ab	41.3ab	20.0ab	27.0b
*Rk1Rk2*	STNCB 2-28	67.5a	55.0ab	36.7ab	16.0bc
*Rk1Rk2*	BAG 29-15-3-32-1	40.0c	36.3b	20.0ab	15.0bc
*N. repanda*	81-R-617A	20.0d	37.5b	3.7b	6.2c

**Notes:** ^*^Data presented are non-transformed means from three, five, four and four replications respectively, inoculated with 5,000 *M. arenaria* eggs. Data were transformed (arcsine) prior to analysis of variance. Means within a column followed by the same letter(s) are not significantly different according to Fisher’s LSD test (*p* ≤ 0.05).

Significant differences in egg mass counts were found among entries in all trials (Table 2). Entry 81-R-617A had significantly fewer egg masses than all entries except for susceptible Hicks in the June to August trial in Blackstone, and fewer egg masses than all entries with the exception of BAG 29-15-3-32-1 in the test conducted in Blackstone from September to November (Table 2). In the trial conducted concurrently in Blacksburg, 81-R-617A had significantly fewer egg masses present than all entries with the exception of CC 13 and STNCB 2-28 (Table 2). In contrast, egg mass production was significantly less on Hicks and K326 than on STNCB 2-28, while egg production on 81-R-617A and BAG 29-15-3-32-1 was intermediate, along with T-15-1-1 and CC 13 in the trial conducted from June to August in Blacksburg (Table 2). Egg mass production was significantly lower on 81-R-617A than Hicks in both trials conducted from September to November (Table 2).

**Table 2.** Egg masses and eggs per gram of root, as well as reproductive indices of *Meloidogyne arenaria* race 2 on flue-cured tobacco entries in greenhouse pot tests in 2017^*^.

		June-August		September-November	
Genotype	Entry	Blackstone	Blacksburg	Blackstone	Blacksburg
*Egg masses per gram of root*					
*rk1rk2*	Hicks	64ab	38b	51a	21a
*Rk1rk2*	K326	90a	41b	45a	22a
*rk1Rk2*	T-15-1-1	83a	86ab	17a	15ab
*Rk1Rk2*	CC 13	89a	98ab	38a	9bc
*Rk1Rk2*	STNCB 2-28	65a	126a	24a	10bc
*Rk1Rk2*	BAG 29-15-3-32-1	67a	60ab	16ab	15ab
*N. repanda*	81-R-617A	30b	60ab	6b	5c
*Eggs per gram of root*					
*rk1rk2*	Hicks	5,697ab	9,938ab	2,907a	439ab
*Rk1rk2*	K326	11,612a	2,728c	1,488a	596a
*rk1Rk2*	T-15-1-1	6,585ab	4,613c	965a	596a
*Rk1Rk2*	CC 13	9,071a	8,311ab	1,197a	170b
*Rk1Rk2*	STNCB 2-28	6,656ab	13,568a	904a	294ab
*Rk1Rk2*	BAG 29-15-3-32-1	5,215ab	7,354ab	946a	268ab
*N. repanda*	81-R-617A	3,988b	5,381abc	17b	54c
*Reproductive index* ^**^					
*rk1rk2*	Hicks	37.9a	3.9c	6.9a	2.1ab
*Rk1rk2*	K326	40.5a	2.3c	4.6a	3.0a
*rk1Rk2*	T-15-1-1	33.4a	6.9bc	3.8a	2.7a
*Rk1Rk2*	CC 13	64.0a	22.9ab	5.5a	0.9c
*Rk1Rk2*	STNCB 2-28	45.1a	26.7a	2.9ab	1.2bc
*Rk1Rk2*	BAG 29-15-3-32-1	36.3a	15.4abc	3.6a	1.1bc
*N. repanda*	81-R-617A	29.3a	6.3bc	0.0b	0.3d

**Notes:** ^*^Data presented are non-transformed means from three, five, four and four replications respectively, inoculated with 5,000 *M. arenaria* eggs. Data were transformed (log_10_ + 1) prior to analysis of variance. Means within a column followed by the same letter(s) are not significantly different according to Fisher’s LSD test (*p* ≤ 0.05).

^**^Reproductive index = final population/initial population (*P*_*f*_/*P*_*i*_).

Significantly fewer eggs were recovered from 81-R-617A than K326 and CC 13 in the trial from June to August in Blackstone, while in the trials in Blackstone and Blacksburg from September to November, 81-R-617A experienced significantly less egg production than all other entries (Table 2). In the trial conducted from June to August in Blacksburg, significantly fewer eggs were recovered from K326 and T-15-1-1 than all entries except for 81-R-617A, which was intermediate in egg production in this trial (Table 2). Entries possessing both resistance genes almost never differed significantly in egg production from whatever entry experienced the greatest egg production in all four trials (Table 2). Egg production was significantly lower on 81-R-617A than Hicks in both trials conducted from September to November (Table 2).

Trends in reproductive indices were similar to those in egg production. Entry 81-R-617A had a reproductive index significantly less than all other entries except for STNCB 2-28 in the trial conducted in Blackstone from September to November, while in the concurrent trial conducted in Blacksburg, the reproductive index of 81-R-617A was significantly less than all other entries (Table 2). In the Blackstone trial at this time, the reproductive index of 81-R-617A was 0.0; reproduction was essentially undetectable, with only 17 eggs present per gram of root (Table 2). Hicks and K326 had reproductive indices significantly lower than entries CC 13 and STNCB 2-28 in the trial conducted in Blacksburg from June to August, and T-15-1-1 had a lower reproductive index than CC 13, while reproduction on 81-R-617Aand BAG 29-15-3-32-1 was intermediate (Table 2). No significant differences in reproductive indices were observed among entries in the trial in Blackstone from June to August (Table 2). 81-R-617A had significantly lower reproductive indices than Hicks in both trials conducted from September to November (Table 2).

## Discussion

The results of these trials suggest that entry 81-R-617A, which possesses traits derived from *N. repanda*, may exhibit resistance to *M. arenaria* race 2 that is greater than that imparted by resistance genes *Rk1* and *Rk2* alone or in combination. Despite some inconsistency in our results, galling was almost always numerically lowest on 81-R-617A, and in two trials this trend was significant relative to all other entries. In three of the four trials, galling on 81-R-617A was significantly lower than that on the susceptible control, and the same pattern significant with respect to reproduction in two trials. However, in one trial, susceptible entry Hicks and K326, which possesses *Rk1* only, had a significantly lower reproductive index than that of entries CC 13 and STNCB 2-28 (which possess both *Rk1* and *Rk2*), while the reproductive index of 81-R-617A was intermediate. In contrast to the observation of Ng’ambi et al. (1999a), we did not see root necrosis associated with reduced egg production in the highly susceptible entry, Hicks, or any other entry; in fact, egg numbers were high on susceptible entries in all four tests. It is unclear exactly what factors contributed to the considerably lower reproduction in both trials conducted from September to November. Inoculum was clearly viable in all four trials based on galling of the susceptible entry, and the inoculum appeared consistent with the initial population from trial to trial based on the morphology of perineal patterns on females recovered from select infested roots. Perhaps differences in daylength and fluctuation between day and night time ambient temperatures were more significant than anticipated.

Way (1994b) demonstrated that a male sterile hybrid, RK3, which is homozygous for *Rk1* and heterozygous for *Rk2*, showed significant reductions in parasitism relative to a susceptible control. This is similar to our observation that entry CC 13, which possesses a similar combination of root-knot nematode resistance genes as RK3, experienced significantly less root-knot nematode parasitism and reproduction than the susceptible entry, but did not differ significantly from the two entries homozygous for both genes, STNCB 2-28 and BAG 29-15-3-32-1, with the exception of one trial in which BAG 29-15-3-32-1 exhibited significantly lower root galling that both CC 13 and STNCB 2-28. However, our results with *M. arenaria* race 2 appear to contrast with Way’s (1994a) observation that BAG line plants, of which STNCB 2-28 is a parent, are significantly more resistant to *M. javanica* than STNCB 2-28, and that BAG lines are more resistant than RK3. We did not detect significant differences in any metrics of reproduction among any of the entries possessing both resistance genes, regardless of zygosity, except in the aforementioned case of root galling.

Resistance to *M. javanica* in male sterile hybrids between *N. repanda* and *N. longiflora* or *N. palmeri* was reported by Schweppenhauser et al. (1963). Davis et al. (1988a) observed a high level of resistance to *M. javanica* and *M. arenaria* in a *N. repanda* line called 46-G, but did not indicate what race of *M. arenaria* was used in their trials. While we saw significant reductions in galling and reproduction on 81-R-617A relative to a susceptible entry and other entries lacking the *N. repanda* derived traits, considerable galling and egg production still occurred in several trials. Davis et al. (1988a) reported that the *N. repanda* entry in their study, 46-G, exhibited little or no gall development relative to standard cultivars; reproduction was inhibited completely, as inferred by the absence of juvenile nematodes in soil at the end of trials (Davis et al., 1988a). The authors also identified sources of resistance to both *M. javanica* and *M. arenaria* in breeding lines from other *Nicotiana* species, but noted that lines with favorable agronomic traits relative to standard cultivars lost this resistance, or tolerance in many cases, upon backcrossing with *N. tabacum* (Davis et al., 1988a). Bui et al. (1992) demonstrated that resistance to *M. arenaria* and tobacco mosaic virus could be integrated into *N. tabacum* from *N. repanda* using a form of protoplast fusion and *N. sylvestris* as a bridge species, but found that nematode resistance was considerably more difficult to retain in backcrosses than resistance to the virus, being present in only 25 of 270 backcrosses. Again, the authors of this study do not indicate what race of *M. arenaria* was used to evaluate root-knot nematode resistance (Bui et al., 1992). Ng’ambi et al. (1999b) observed a ‘moderate’ level of resistance to *M. arenaria* race 2 in 81-R-617A and a related breeding line, 81-RL-2K, in contrast to a ‘high level’ of resistance observed in a breeding line from South Africa, SA1214. In their trials, an average of over 7,000 *M. arenaria* race 2 eggs were present per gram of root for entry 81-R-617A, compared with fewer than 3,000 egg per gram of root on SA 1214, a considerable difference, but one that is not clearly identified as significant. Importantly, the authors note that these lines are the first *N. repanda* entries to be identified with resistance to *M. arenaria* race 2 that could be compatible with existing *N. tabacum* accessions for breeding and crop improvement (Ng’ambi et al., 1999b).

Ng’ambi et al. (1999a) demonstrated that resistance to *M. arenaria* race 1 in a breeding line related to 81-R-617A, 81-RL-2K, is conditioned by the same single-dominant gene which confers resistance to *M. incognita* races 1 and 3 in the commercial flue-cured tobacco cultivar Speight G 28. This gene, *Rk1*, is widely available in commercial flue-cured tobacco in the United States (Koenning et al., 1999). *Rk1* confers resistance to root-knot nematodes via a hypersensitive response that inhibits nematode feeding site development and subsequent galling and reproduction (Ng’ambi et al., 1999b; Schneider, 1991) The presence of this gene explains the resistance to ‘*M. arenaria*’ populations not identified to race observed by other authors, which can be inferred to have been race 1 (Bui et al., 1992; Davis et al., 1988a). However, *Rk1* does not impart resistance to *M. arenaria* race 2, and appears to have relatively little impact on *M. javanica* (Ng’ambi et al., 1999b), suggesting that a different system is responsible for the resistance to these nematodes observed in *N. repanda* and related breeding lines.

Another resistance gene, known as *Rk2*, provides considerably greater resistance to *M. javanica* than *Rk1,* and if the two genes are ‘stacked’ an even greater level of resistance is conferred (Ma et al. unpublished data; Ternouth et al., 1986). There is also evidence that the combination of both genes confers a significantly greater degree of resistance to a variant of *M. incognita* race 3 than either resistance gene alone. In the study in question, *Rk1*, rather than *Rk2*, was associated with greater reductions in nematode parasitism and reproduction (Pollok et al., 2016). The mechanism of resistance associated with *Rk2* is unknown, although it has been speculated to be different than the hypersensitive response associated with *Rk1* because *Rk2* does not appear to consistently inhibit gall formation, but does reduce reproduction (Pollok et al., 2016).

It is unclear what gene or genes interact with *Rk1* to confer the relatively high level of resistance to *M. arenaria* race 2 we observed in the line with *N. repanda* traits in our trials, which was equivalent to or greater than that exhibited by entries possessing both *Rk1* and *Rk2*. The relatively drastic reductions in root galling we observed on 81-R-617A suggests the possibility of a hypersensitive response. In tomatoes, diminished nematode parasitism has been associated with the inhibition of penetration due to alterations in the composition of root exudates perceived by infective juveniles in the presence of arbuscular mycorrhizal fungi (Vos et al., 2012). Augmentations of root morphology have been observed in nematode resistant *Prunus* spp., wherein resistant cultivars feature a different arrangement of epidermal cells in root tips compared with susceptible cultivars (Ye et al., 2009). Pollok et al. (2016) speculated that *Rk2* may simply slow down nematode feeding and development, or is involved in inhibition of a specific stage of egg mass development. It is possible that some combination of the aforementioned mechanisms is involved in resistance to root-knot nematodes in flue-cured tobacco entries like 81-R-617A that possess traits derived from *N. repanda*.

It would be of great value to determine what gene or genes are implicated in nematode resistance in *N. repanda*. As Ng’ambi et al. (1999a) observed, the genetic basis of resistance associated with *Rk1* is ‘narrow’ and they cautioned that ‘repeated use of this resistance may select resistance-breaking biotypes.’ As Murphy et al. (1987) observed, tobacco cultivars within similar market classes are highly related. This presents potential challenges for breeders attempting to improve disease resistance and agronomic traits in cultivars in the future because of a limited genetic diversity within available germplasm (Lewis and Nicholson, 2007). Introducing new genetic sources of disease resistance to this pool of germ plasm from sources like *N. repanda* could help remedy some of these challenges. Additionally, moving forward, tobacco growers in the southeastern United States face a new, highly destructive, polyphagous nematode threat in the form of *M. enterolobii*. This nematode, which has recently been found on root-knot resistant sweet potato in South Carolina (Rutter et al., 2019) and is present in at least eight counties in North Carolina (Schwartz, 2019) is a quarantine-level pathogen (Thiessen, 2018) that, while not yet known to be directly impacting tobacco growers, presents a potentially serious threat, as this nematode is not controlled by available forms of resistance including *Mi* and *Rk*-mediated resistance (Ye, 2018). Perhaps exploiting *N. repanda* germplasm, which has long been a source of novel traits for cultivated tobaccos (Stavely et al., 1973) could present solutions to these and other issues facing tobacco breeders and growers alike.
